# Improving Cancer Statistics in the United States

**Published:** 1916-10

**Authors:** 


### Abstracts and Selections.

### Improving Cancer Statistics in the United States.

At the suggestion of a number of the foremost American students of the cancer problem, the United States Bureau of the Census has instituted radical improvements in the collection and publication of the statistics of this disease. A special report on deaths from cancer in the United States during the year 1914 is in preparation and will be issued shortly after the first of the year. Inquiries recently received by the Director of the Census having indicated that some misapprehension has arisen in regard to the purpose and scope of this study, the American Society for the Control of Cancer has issued a statement explaining the significance and essential features of this project of the Census Bureau which is thought not only to promise important additions to our knowledge of cancer but to constitute a noteworthy advance in the registration of American vital statistics.

It should first be made clear that the Census Bureau has not undertaken special research work that will in any way duplicate the studies of existing institutions and laboratories which are investigating the cause of cancer. On the other hand, there should result a marked improvement of our national mortality statistics of this disease in the direction of greater accuracy and more detail. The experience of foreign countries has shown the value of perfecting and carefully analyzing the annual statistics of deaths in order to throw new light upon the cancer problem, which still remains the chief outstanding question in the realm of medical science.

In February, 1914, the American Society for the Control of Cancer suggested to the Federal authorities that the figures of deaths from cancer in the United States Registration Area be published in greater detail, and that instead of being reported under only seven headings, as had been the custom hitherto, they be listed under many more titles according to the part of the body first affected, thus affording opportunity for more exact comparative study. The suggestion received favorable consideration by the Bureau of the Census, and a special report for 1914 was ordered begun by the former director, Hon. William J. Harris, and is now nearing completion under his successor, Hon. Sam L. Rogers. This special monograph on cancer will consist of tables showing the deaths from cancer, according to the site of the disease, age, sex, color, nativity and marital condition, for the registration area, the several registration States and the usual subdivisions. Figures for white and colored will be shown separately for such counties and towns as have a colored population of 10,000, or at least 10 per cent of the total. The new plan subdivides the seven titles for cancer in the International List of the Causes of Death into twenty-nine headings referring to the exact site of the disease. For instance, all deaths from "cancer and other malignant tumors of the buccal cavity" will now be reported under the separate subdivisions for cancer of the lip, tongue, mouth and jaw, and similarly with the other groups.

Upon the further suggestion of a prominent surgeon the Census Bureau also planned to increase the accuracy of the statistics by tabulating separately the returns in which the diagnosis was "reasonably certain" and those in which it was "uncertain." In arriving at this distinction a report is classed as "certain" if the diagnosis was confirmed by microscopical examination of tissues, or by surgical operation, or by autopsy. All cases of internal cancer in which the diagnosis was based on clinical observations alone are classified as "uncertain" regardless of any strength of assertion by the physician that the diagnosis was correct. At the request of the Census Bureau an advisory conference, including representatives of the Harvard Cancer Commission, the George Crocker Special Research Fund of Columbia University, the Barnard Free Skin and Cancer Hospital of St. Louis, the New York State Institute for the Study of Malignant Disease, the Prudential and Metropolitan Life Insurance Companies, the American Association for Cancer Research and the American Society for the Control of Cancer, considered the details of the plan and assisted in the formulation of instructions for editing certificates of deaths from cancer in preparation for the special report. To gather the necessary detailed information the Director of the Census has sent over 35,000 letters of inquiry to physicians who certified deaths from cancer during 1914.

Although a large amount of additional labor has been thrown upon the Division of Vital Statistics of the Census Office by the preparation of this report, it is believed that the trouble and expense will be more than repaid by the result. The improvement of cancer statistics has practical bearings of greater consequence than may at first appear. Indeed, the importance of statistical investigation in arriving at the solution of the cancer problem is likely to be overlooked. Much of the valuable knowledge of the disease which we possess today has resulted from the collection and comparison of statistical data, and this method must be relied upon, side by side with experimental research and clinical observation, to elucidate the baffling problem of the nature and cause of this disease. The publication of this report by the Census Bureau should bring out new and useful information as to the prevalence of the disease in the United States and thereby contribute to the better understanding of its controllable features. Such a study as the Census Bureau is making, if continued, should also throw clearer light on the question of whether or not cancer is really increasing. The foremost authorities have repeatedly urged that this question can be scientifically answered only by studying separately the facts in regard to each of the many forms and sites of malignant disease. The Imperial Cancer Research Fund has co-operated with the Registrar-General of England and Wales in a thorough analysis of the detailed figures for cancer of the stomach, cancer of the tongue, cancer of the breast, etc., for successive years. By the progressive action of the Director of the Census similar data as to parts of the body affected on which such studies can be made will now become available for the first time in the official statistics of the United States.

The new plan will not only produce data for the year 1914, but every future year a vast amount of information will be recorded and stored away, and can be compiled and published when the demand warrants. Efforts are also being made further to co-ordinate the work of the State and Federal statistical offices for the better registration of deaths from cancer and other diseases as well. By the operation of this plan and the mutually supplementary efforts of the national and State registration officials, it will be possible permanently to record and study the extensive American data on cancer mortality, with all the detail required by the most exacting statistical methods.—*Courtesy of Press Service of the American Society for the Control of Cancer.*

